# The Eurostack of digital health cybersecurity

**DOI:** 10.3389/fdgth.2026.1894369

**Published:** 2026-08-11

**Authors:** Celia Brightwell, Chiara Carboni, Orit Halpern

**Affiliations:** 1 Chair of Digital Cultures, School of Humanities and Social Sciences, Technical University of Dresden, Dresden, Germany; 2 Department of Technology, Management and Economics, Division of Technology and Business Studies, Technical University of Denmark, Lyngby, Denmark

**Keywords:** Big Tech, cybersecurity, digital health, digital sovereignty, Eurostack, state-platform

## Abstract

Cybersecurity infrastructures are designed to mitigate the risks posed by two common entities: the layperson who circumvents cybersecurity guidelines, and the malicious actor who exploits weaknesses in security mechanisms. In this perspective, we show that a third entity, the US state-platform, is re-shaping cybersecurity infrastructures for digital health in Europe. The state-platform describes the centralization of power signified by the close relationship between the second Trump administration and the rise of the tech oligarchy. Drawing on interviews with experts working on cybersecurity for digital health in Europe, we show how concerns about the state-platform are driving digital sovereignty interventions consistent with the Eurostack initiative.

## 1 Introduction

Cybersecurity infrastructures for digital health systems in Europe are designed to mitigate the risks posed by two common entities. First, the layperson who mistakenly or intentionally [often with due cause (1)] circumvents cybersecurity guidelines. Second, the malicious actor, existing in the cultural imaginary as a Russian or Chinese hacker (2, 3), who exploits weaknesses in security mechanisms. In this perspective paper, we present a viewpoint that a third entity is re-shaping cybersecurity infrastructures for digital health in Europe: the US “state-platform” (4). The state-platform describes the close relationship between the second Trump Administration and the rise of what legal scholar Julie Cohen terms a tech oligarchy, as captured in the infamous photo of figures including Jeff Bezos, Elon Musk, and Mark Zuckerberg at the 2025 presidential inauguration (4). As an entity, the state-platform: claims extraterritorial reach over data even when stored in Europe; threatens tariffs against countries that seek to regulate US tech firms; and drains fledgling European alternatives of talent and capital (5). Amidst the growing scholarly discourse on digital sovereignty, this is prompting concerns regarding “Europe’s ability to act independently in the digital world” that have been central at least since the Covid-19 pandemic (6). In her mapping of the network of contracts and funding relationships connecting tech oligarchs with the Trump administration, economist Francesca Bria warns: “tech billionaires are building a post-democratic America” and “Europe is next” (7).

The question of digital sovereignty, which has become a cornerstone for policies in the EU and beyond, materializes in digital health with life-and-death stakes. The potential implications of failures in digital health, including cybersecurity breaches (8, 9), mean that the field's cybersecurity experts must be hyper-vigilant to new forms of risk, anticipating the more general risks posed by what Bria terms “technological imperialism” (5).

This perspective draws on original data from eight semi-structured interviews, each lasting approximately one hour, with experts involved in European cybersecurity for digital healthcare. Participants were recruited through our professional networks as researchers involved in an EU project addressing cybersecurity in the internet of medical things. Inclusion requirements concerned participants' current work on cybersecurity in Europe's medical sector. Our respondents include practitioners in hospital information security teams and academic research institutes, software engineers from small and medium enterprises, medical device regulatory scientists, and connected medical device manufacturers. Amidst the rapidly evolving geopolitics we outline in the next section, this small interview sample size and European-only perspective – our respondents work on cybersecurity in medicine in Italy, Portugal, Switzerland, and Germany – mean the interview data component of this perspective functions as a snapshot rather than offering widespread generalizability. Data was coded abductively to integrate empirical insights with extant literature while staying open to emerging themes (10), jointly performed and discussed by the first two authors to guarantee inter-rater reliability. The combination of these qualitative analytical strategies – close reading of empirical data, contextualization with existing research, and ongoing hermeneutical dialogue within the team – allowed us to moderate and reflect on the bias that, as situated actors, we inevitably bring as researchers.

We argue that, in the current geopolitical climate, the question of responsibility for good patient care increasingly intersects with questions related to the security of digital infrastructure. Ultimately, in the current geopolitical juncture, it is concerns about the US state-platform that disproportionately reshape “the stack” of European digital health.

## 2 The Eurostack

In an open letter calling for sovereign digital infrastructure, Eurostack signatories cite developments in US–EU relations to describe an acute moment of crisis for security and strategic autonomy (11). The “stack” is a term philosopher Benjamin Bratton coined that connotes a megastructure made up of interlocking layers of hardware, software, networks and data (12). Bria uses the stack to describe “the operating system of modern political and economic power,” noting that while geopolitical power once flowed through “armies and treaties,” today it “courses through silicon wafers, server farms and algorithmic systems” (5). The digital health stack, made up of the servers, systems, and software that facilitates everything from hospital-at-home settings to personal fitness wearables, is inextricable from these geopolitical tensions.

Those advocating for a Eurostack position the initiative as necessary based on the reality of US–EU relations but emphasize an affirmative vision of the digital future for Europe. This is one buoyed by democratic values and robust regulation, and one in which digital technology serves social, economic, and ecological goals. In highlighting that not only digital health but governments, businesses, and citizens in Europe depend on American infrastructure designed and governed to serve American interests, the Eurostack initiative crystallizes what is presented as a growing fissure between the interests and values of the US and those of Europe. To illustrate the impetus behind the project, Bria writes: “One executive order in Washington – not Brussels or Berlin – could cut access to critical systems running our industries, hospitals and elections” (5). Referring to Trump's threats to ban and sanction European regulators who implement digital laws that limit the reach of Big Tech in Europe, she suggests that while regulation remains a meaningful obstacle to what she terms “US technological supremacy,” what is really needed is “infrastructure sovereignty” (5). In its current state, the Eurostack is backed by 200 businesses, endorsed in France and Germany's national strategies, and comprises cloud storage (OVHcloud, Nextcloud), communications (Proton), and AI models (Mistral) (5). Contributing to this nascent Eurostack assemblage is infrastructure in development for the cybersecurity of digital health networks in Europe.

The digital sovereignty agenda is not without its critics. Bratton, for instance, has argued that the Eurostack constitutes a too-late, misdirected and futile reaction to the centralization of geopolitical and infrastructural power that the US and China have been driving for decades (13). Media studies scholars Grohmann and Costa-Barbosa have noticed that the term has increasingly turned into an empty signifier, amenable to being captured by the very platform companies that it attempts to escape (14). The digital sovereignty agenda's emphasis on geopolitical regions such as Europe might also detract from other possible “stacks,” such as those based on economic models most compatible with public healthcare. New stacks might address existing economic problems of liquidity, value, and distribution so that they do not respond to the problems posed by US Big Tech by creating another European oligarchy.

Despite digital sovereignty's current political prominence, it is also worth noting how Big Tech's encroachment into more spheres of social and public life has established forms of power and dominance that go well beyond the scope of digital sovereignty (15). For instance, philosopher Marjolein Lanzing suggests that as software such as Palantir comes to constitute parts of the stack in fields as disparate as warfare and healthcare, there is a risk that the “sphere of health will increasingly be structured according to the industrial logic and values of the sphere of security, crowding out those of care, changing its meaning” (16). In a time in which geopolitical instabilities intersect with infrastructural (inter)dependencies, the boundaries between different spheres become blurred, with geopolitical considerations being increasingly part of healthcare infrastructure-building, and the very meaning of care being stretched to accommodate emerging concerns.

### 2.1 Technological imperialism

The themes of US technological dominance and imperialism that Bria mobilizes to argue for a Eurostack also surfaced in our interviews. Palantir emerged as perhaps the most blatant example of American software signaling American interests:

“Palantir's core business is absolutely that the US should be protecting their interests through leading the world: world domination in information and data processing” (interview seven).

This statement represents perceptions regarding how US technological imperialism is impacting digital health in Europe that characterized the topic of geopolitics among participants. Geopolitical insecurity, including the developments between US–EU relations that Eurostack signatories describe, exacerbates cybersecurity concerns. This is most obvious in the fact that, as one participant noted, “in wars cybersecurity attacks also increase, these are concerns of the wars” (interview one). Typical tropes of malicious actors representing foreign interests reinforced the connection between national and digital health security:

“You need to act as a nation in some things. I can tell you for example that most of our attacks are from Russia, from other countries, so there is a need of going beyond the simple local site level and escalating towards regional, national, and political levels, because now these matters are this big” (interview three).

“If you’ve got a situation where one country is in a major global conflict with another, would they do this [hack connected implanted medical devices such as pacemakers]? Well, of course they would” (interview seven).

We suggest that the US-state platform emerges as a new form of risk: not as the “attacker” or hacker alluded to in these examples, but rather as one operating through the scale afforded by technological imperialism. One participant noted that the tension between medical device security and national security is complicated by the slippage between general consumer devices such as smartphones, which are inextricable from medical devices such as apps and connected wearables. This function creep means consumer products are the medical devices of the future, which significantly expands the remit of cybersecurity concerns for medicine. For example, a “back door” introduced accidentally or intentionally into the hardware of a device can be a means of compromised information security:

“Are people introducing back doors into consumer products for state control? Yes… Apple was made to introduce a back door to their highly secure iCloud by the UK, but then the UK was made to stop that, not legally, but because Trump said you’re not doing that as part of the trade negotiations, and they dropped it. And they're trying to bring it back in but just for British citizens, so there'd be a back door to the Apple cloud data” (interview seven).

That smartphones can be considered medical devices of the future highlights the challenges inherent to a digital health Eurostack. However, focusing on two layers of the stack, cloud storage and AI, we show how procuring or developing European alternatives is already shaping digital health cybersecurity infrastructures.

### 2.2 Cloud storage

That “the cloud” is a fuzzy misnomer connoting an ethereal, floating capacity becomes apparent in expert perspectives on the geopolitics of cybersecurity infrastructure. Cloud storage relies on physical servers that are hosted in specific and strategic geographic areas. Participants highlighted the affordances of the cloud as a centralized data storage option that facilitates interoperability and data exchange for research purposes. For instance, cloud storage can more easily function as a central platform to connect and anonymize data from thousands of hospitals, enabling AI-driven disease detection. However, participants lamented the lack of a viable cloud solution in the EU, which would reduce dependence on US hyperscalers – namely Amazon, Google, and Microsoft. While participants acknowledged products from Big Tech to be very good in terms of user experience, interoperability, and data exchange, they demonstrated a lack of trust in these systems and a desire for European alternatives. Participants discussed the choice to use or not to use the Amazon Web Services (AWS) cloud platform in terms of data sensitivity:

“I would not have chosen AWS if I had patient PII [personally identifiable information], because then you enter in different conversations, it's probably better to choose a provider that's hosted in Germany, but where the US privacy rules, or lack of privacy rules, don't apply, then I probably would have chosen a different technology, that is more a sovereign cloud” (interview two).

“Yeah, I don't use AWS, I'm using a cloud provider, so when we talk about supporting projects in which the servers would be on European soil.. If we look into what would be a realistic deployment for an extremely conservative customer, it would be on the [hospital] premises” (interview six).

This reflects both the participants' perspective that Europe has some of the world's more stringent data protections [for example, “If it's not European… then there could be some concerns on how the data is shared and exposed” (interview six)], and the sense of mistrust towards non-European providers. Other participants reflected a concern about being “locked in” as a customer to dominant platforms:

“Some companies like AWS or Microsoft, basically cloud platform providers, are primarily interested in making sure that their customers cannot hack their systems. They may not necessarily have it as a first priority to secure customer workloads because if they [the customers, such as IT service providers] have no choice, they will still give them money” (interview four).

Consistent with the Eurostack narrative, the alternative to US providers such as AWS is to develop a sovereign cloud: a slow, expensive, and technically difficult process. One participant described setting up a server on a healthcare client's premises, which must then be monitored for data availability and incurs additional expenses, such as for electricity and maintenance. The physical security infrastructure surrounding cloud server locations – fences, alarms, human security officers – also shapes the practices and imaginaries of cybersecurity in digital health.

Developing a sovereign cloud alternative is positioned as an option that offers additional security due to less exposure of data to third parties, and is also a value proposition that small European cybersecurity firms can offer to clients:

“But now we talk about sovereignty, you know it has a cost. So it would be cheaper to go to this provider, but then I would be depending on this provider. If I [were] relying on connected devices, so to speak, from companies which already have the whole ecosystem implemented I would not have to do it myself, but I would lose control of the data. And also, what would be my added value?” (interview six).

While these participants consistently described the need for increased European digital sovereignty, in the short term this was acknowledged to be challenging in practice. For instance, taking into account the broad network of connected devices involved in digital health systems, this would involve finding open-source or European technologies that include not only clouds and servers but also operating systems, databases, computer chips, and AI models.

### 2.3 AI

The increasing pervasion of AI in healthcare systems introduces a new scale to the risk-benefit trade-offs of digitalization in healthcare – trade-offs that are inflected by the anticipation of the US state-platform as a new form of risk.

AI is already widely used in digital health systems. As one participant noted, “[We had to ban] access to ChatGPT from our network, because physicians started to put patient data into ChatGPT. It was quite natural for them” (interview three). In an example of a micro Eurostack intervention, the IT team built its own on-premises LLM for physicians to use instead. The team concedes that their LLM is not as good as US leaders such as ChatGPT and Gemini, but that they were obliged to build it to preserve patient privacy.

Participants characterized AI as increasingly central, both as threat and asset. Insofar as AI is used in attacks, it becomes necessary for defence, and participants anticipate a future of “AI versus AI” (interview six), in which systems autonomously attack and defend the cybersecurity of Europe's digital health infrastructures. For example:

“Now there are a lot of threats coming automatically from AI agents, they build them on purpose and attack you…Being so up to date that we will be able to face these kind of threats, it's not easy, not easy at all, in terms of computational power, AI is a giant, and it's very likely that we will face something we've never seen before” (interview three).

As one participant noted, the capability of AI to identify specific behavior patterns and flag a potentially malicious use case is nothing new: this is how virus scanners have already been operating for a long time (interview four). This participant was circumspect about the usefulness of AI to cybersecurity engineering, highlighting that “security” is “about being correct and exact, and those models are the opposite, they are not exact” (interview four). However, if the AI model is, according to this participant, less correct and exact than a human cybersecurity engineer, they concede that AI is faster and, in their words, more “creative” in its ability to find new weaknesses in cybersecurity, and that this means AI is “shifting who is ahead in the arms race between attacks and defences” (interview four).

Despite emphasizing the risks AI presents to the security of digital health systems, participants overwhelmingly expect that “in the healthcare sector the use of AI has more pros than cons” (interview one). While the US is ahead in the AI race, as our participant whose team built an alternative LLM suggested, European cybersecurity experts are willing to invest time, expenses, and expertise to build sovereign alternatives.

## 3 Conclusion

The sovereign LLM and the cloud storage server that participants built are evidence of how the tide of mistrust towards the state-platform is materially shaping Europe's digital health infrastructure. Teams in Europe are building sovereign technology despite considerable commercial, technological, and time constraints, and despite conceding that, in the case of the LLM, the result is “not as good,” or at least “not as capable of the wonderful performances” of US leaders such as ChatGPT. Healthcare cybersecurity experts display a striking awareness of Big Tech power, consistently casting the US state-platform as a threat. There is a sense that when European technologies gain traction, it is difficult to compete in the global market given US dominance in the field. Bria describes the “steady drain” of talent and capital once European researchers and tech startups attract the attention and funding of Silicon Valley, and lawsuits bankrolled by US tech giants that follow European cities asserting digital sovereignty (5). The Eurostack initiative seeks to address this, for instance by creating a sovereign investment fund to support the development of European technology (17).

As cybersecurity becomes an increasingly central concern for public organizations and healthcare systems, protecting security and European interests come to determine which technologies are used in digital health systems, potentially outweighing considerations such as expense and user experience. This, as we have seen, happens by way of a reframing of the notion of care and responsibility in the digital health context. Here, responsibility becomes increasingly not only about taking care of patients, but also of digital infrastructures (18) – a task made all the more complex by current geopolitical instabilities. IT teams are also performing a care function, not exclusively an imposition of security logics (1).

In one sense, the Eurostack project delineates an end to the 1990s techno-utopian dreams of digital technology fostering a democratic global community by overcoming geopolitical boundaries. However, by emphasizing “openness” as a pillar of Europe's digital sovereign strategy, it retains a kernel of these early possibilities and identifies a path forward from what is today described as a broken internet (19, 20). Grassroots development of LLMs and cloud storage servers cannot take on Big Tech alone, but this is evidence that there is a rising tide of those taking action to do so. Crucially, this testifies to how rethinking technology ownership models beyond the oligarchic ones propagated by the state-platform must be a central point of any debate around digital sovereignty.

## Data Availability

The anonymized raw data supporting the conclusions of this article will be made available by the authors, without undue reservation.
